# Regulation of glucose homeostasis by calorie restriction and periodic fasting

**DOI:** 10.18632/aging.104217

**Published:** 2020-12-13

**Authors:** Volha Mezhnina, Roman Kondratov

**Affiliations:** 1 BGES Department and Center for Gene Regulation in Health and Disease, Cleveland State University, Cleveland, OH 44115, USA

**Keywords:** aging, metabolism, insulin sensitivity, circadian rhythms, feeding

Increased blood glucose, impaired glucose tolerance and reduced insulin sensitivity are highlights of metabolic syndrome and type two diabetes. Glucose homeostasis is also frequently disrupted in cancer, cardiovascular diseases and neurodegeneration. Disruption of glucose homeostasis is also associated with age and aging significantly increases the risk of above diseases. Interventions that improve glucose metabolism might be beneficial for treatment of various pathologies. Diet is one of the most common ways to affect glucose homeostasis, and various dietary interventions have grown in popularity as complementary strategies to prevent and manage diseases associated with metabolic disorders. For roughly a century, caloric restriction, a reduced intake of calories without malnutrition, has been reported to induce multiple health and longevity benefits across taxa [1]. Reduced blood glucose, improved glucose tolerance and increased insulin sensitivity in CR mammals is well documented.

Experimental CR in rodents is associated with periodic fasting. It was speculated that some or even all of the benefits of caloric restriction might be due to periodic fasting. Several diets that explore periodic fasting were proposed as an alternative to CR. Time restricted feeding (TRF) is one diet that implements periodic fasting and has recently gained significant public attention [2]. In a TRF diet, *ad libitum* (AL) feeding is allowed during a restricted period of time. TRF was recently explored as a therapeutic intervention in pre-clinical trials. In humans, 6-hour TRF provides some benefits, however, the benefits might be due to a concomitant reduction in caloric intake [3], and no significant benefits were reported in other studies that did not reduce caloric intake [4]. From a practical point of view, TRF might be a more convenient diet for humans than CR; indeed, time is easier to monitor compared with calories. Disruption in glucose metabolism contributes to many pathologies, and whether TRF can be as effective as CR to manage glucose homeostasis is an important question.

**Figure 1:**
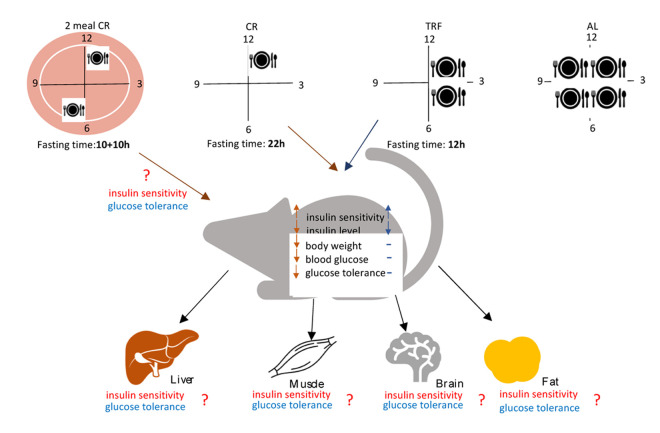
**Caloric restriction and time restricted feeding are popular dietary interventions that implement periodic fasting.** Both diets improve insulin sensitivity, but only CR significantly improves glucose homeostasis. This unexpected uncoupling of highly intertwined insulin sensitivity and glucose homeostasis can be explained through a selective effect of the diets on tissue insulin sensitivity. The diets differ in the magnitude of calories and duration of fasting. Two meal per day CR will reduce the duration of fasting and may elucidate the relative contributions of caloric intake and fasting on glucose homeostasis. (See more details in the text).

To address this question directly, we compared the effects of CR with 12-hour TRF [5]. The food was available AL for 12 hours every day and no reduction in caloric intake was detected. Effect on glucose homeostasis was assayed in healthy young rodents on regular chow. The results of the study are summarized in Figure 1. Surprisingly, while both diets reduce blood insulin and increase insulin sensitivity, compared with AL fed mice, only CR, and not TRF reduces blood glucose and improves glucose tolerance. The study concludes that periodic fasting was responsible for some of the metabolic changes induced by CR, and improved glucose homeostasis requires reduced caloric intake. However, this conclusion must be taken with caution, due to CR and TRF mice fasting for different lengths of time. CR mice consumed all the provided food in two hours, therefore, they fasted for about 22 hours, while TRF mice were fasted for only 12 hours. The duration of fasting may be important and brings another important question: is there an optimal duration of fasting? This may be experimentally testable.

One approach, in attempt to increase fasting time, may be implemented through narrowing the window of AL food availability. At first glance, it is not a complicated task, but it might be challenging or even impossible to design. The reduction in duration of food availability may also reduce the amount of consumed food. A reduction of food availability for only 6-8 hours results in 15-20% reduced food intake for both rodents and humans. Therefore, it might be impossible to achieve continuous 22-hour fasting without reduction in food intake. A complimentary approach is to reduce fasting time in CR, which can be achieved by providing CR daily food as two or more meals per day instead of the traditional one meal a day (Figure 1). This approach will reduce caloric intake without a long continuous fasting. If the duration of fasting is critical for improvement in glucose metabolism, we might expect that multiple-meal CR will not be as efficient as single-meal CR in improvement of glucose homeostasis.

Another interesting question raised by Velingkaar et al. study [5] is a connection between insulin sensitivity and glucose homeostasis. Glucose homeostasis and insulin sensitivity are tightly linked: impaired insulin sensitivity, for example in metabolic syndrome, is associated with a defect in glucose homeostasis, and high insulin sensitivity in CR is associated with improved glucose homeostasis. Therefore, the improved insulin sensitivity without the improvement in glucose homeostasis in TRF is counterintuitive. In addition to glucose, insulin also regulates fat metabolism. High insulin favors carbohydrate oxidation and fat biosynthesis, while low insulin favors fatty acid oxidation. The ability of tissues to switch between different substrates such as carbohydrates or fat for energy production is called metabolic flexibility and requires cooperation of metabolically different tissues [6]. Little is known on the contribution of periodic fasting and reduction in caloric intake on metabolic flexibility of individual tissues (Figure 1). One possible explanation for uncoupling insulin sensitivity and glucose metabolism in TRF is that CR improves metabolic flexibility of all major metabolic tissues (liver, muscles and adipose), and periodic fasting improves metabolic flexibility for only some tissues. Molecular mediators of insulin action in tissues such as insulin receptor or Akt kinase are well studied. Tissue specific knockouts for many genes in insulin signaling are available. Comparing the effects of CR and TRF on glucose homeostasis in tissue specific knockouts will shed light on mechanisms of dietary interventions.

In summary, caloric intake and duration of feeding independently contribute to the effects of diet on physiology. Therefore, to better understand mechanisms of metabolic adaption, both factors must be taken into consideration when the effect of the diet is studied in mammals.
